# Patterns of contact call differentiation in the panmictic East African Abyssinian White‐eye *Zosterops abyssinicus* (Aves: Passeriformes)

**DOI:** 10.1002/ece3.1828

**Published:** 2015-12-09

**Authors:** Jan Christian Habel, Martin Husemann, Werner Ulrich

**Affiliations:** ^1^ Terrestrial Ecology Research GroupDepartment of Ecology and Ecosystem ManagementSchool of Life Sciences WeihenstephanTechnische Universität MünchenD‐85354FreisingGermany; ^2^ General ZoologyInstitute of BiologyMartin‐Luther University Halle‐WittenbergD‐06120Halle (Saale)Germany; ^3^ Chair of Ecology and BiogeographyNicolaus Copernicus University in ToruńPl‐87‐100ToruńPoland

**Keywords:** Acoustic communication, allopatry, character displacement, contact calls, geographic gradients, sonogram, sympatry

## Abstract

Species distribution patterns range from highly disjunct to continuous, depending on their ecological demands and the availability of respective habitats. East African savannahs are mostly interconnected and ecologically comparatively homogenous and thus provide a prerequisite for a rather panmictic distribution pattern for species occurring in this habitat. The Abyssinian white‐eye *Zosterops abyssinicus* is a savannah inhabiting bird species, representing such a continuous distribution. This species occurs in high abundances and is very mobile, and past population genetic studies have suggested that gene flow is high and genetic differentiation is low even across relatively large geographic distances. Further, only little morphological differences were found. In order to test for potential divergence in acoustic traits despite its interconnected geographic distribution, we analyzed 2795 contact calls of *Z. abyssinicus,* which were recorded at 19 sites across Kenya. Our data indicate weak, but significant differentiation in call characteristics across latitudinal gradients. We found strong changes in call characteristics in populations where *Z. abyssinicus* occurs in sympatry with its highland congener, *Zosterops poliogaster*. However, the changes in call characteristics in sympatry were in different directions and lead to strong differentiation of the sympatric populations to other conspecific populations potentially representing a case of cascade reinforcement. The detected spatial gradients likely result from ecological differences and balancing effects of natural and sexual selection.

## Introduction

The extent of a species′ distribution depends on habitat demands, the dispersal behavior of species, the availability of the respective habitat type and local competition (Lomolino et al. 2006). While species with specific habitat demands and limited dispersal behavior often occur in small and isolated habitat patches, species with a broad ecological tolerance and high mobility are often found in interconnected population networks (Devictor et al. 2008). These contrasting distribution settings are often reflected by the intraspecific population structure: studies showed that habitat specialists with disjunct distributions are characterized by strong within‐taxon differentiation and a comparatively low intraspecific variability, while taxa with panmictic distributions often do not show signals of intraspecific divergence, and thus generally have larger intraspecific variability (Hampe and Petit 2005).

Intraspecific differentiation can be driven by a variety of factors: (1) geographic isolation (e.g., isolated mountains) can lead to strong splits within taxa, especially when populations are rather small (Habel et al. 2014; Husemann et al. 2015a), or (2) divergence can be found within one taxon when populations have more than one ecological optimum, e.g. specialization to two different host plants, which may lead to adaptation (Egan and Funk 2009; Funk et al. 2011); Finally, (3) intraspecific differentiation may evolve from premating barriers, which may become enforced by character displacement to avoid cross‐taxon hybridization. Such processes may occur, when species initially occurred in allopatry and subsequently formed secondary contact zones (Brown and Wilson 1956; Schluter and McPhail 1992; Husemann et al. 2014a). Character displacement occur on ecological as well as sexually selected characters, may reduce competition and avoids forming unfit hybrids (Pfennig and Pfennig 2009). If populations occur in sympatry with others and character displacement in reproductive traits is strong and trait shifts are heritable, cascade reinforcement occur, leading to the rejection of individuals of the sympatric population by conspecific individuals of other populations (Ortiz‐Barrientos et al. 2009; Kozak et al. 2015). This mechanism may then lead to increased rates of speciation.

Acoustic traits are commonly suggested to be sexually selected, but may also be affected by the local ecological conditions (Kroodsma and Miller 1996). With the seminal articles by Morton (1975), and Wiley and Richards (1978), Wiley and Richards (1982) and later on supported by empirical work of Hunter and Krebs (1979), the theoretical framework for predicting environmental effects on acoustic signaling behavior was developed. The evolution of bioacoustic characters is driven by various factors, such as geographic differentiation, divergent local selective regimes, sexual selection, selection for species recognition, or a balance of natural and sexual selection (Irwin 2000). Here, we attempt to distinguish between two main drivers: nonselective effects, such as drift (geographic isolation and/or small population sizes) (see Laiolo and Tella 2006), and adaptive processes (any form of selection) (e.g., Irwin 2000; Husemann et al. 2014b; with references therein). For example, analyses of the contact calls of disjunct mountain populations of the East African Montane white‐eye complex (*Zosterops poliogaster* and close relatives) showed distinct mountain specific call patterns, most likely resulting from long‐term geographic isolation (Habel et al. 2013; Husemann et al. 2014b). Similar patterns of genetic divergence pointed toward strong effects of neutral processes (Habel et al. 2013). Likewise, congruent genetic and acoustic patterns were found for the four‐eyed frog (Velásquez et al. 2013), for which the authors suggested a genetic basis of call divergence. In contrast to such geographically driven differentiation patterns, bioacoustic traits may also be affected by local environmental conditions as exemplified by call divergence between urban and rural environments in the House Sparrow and the Grey Tit (Katti and Warren 2004; Warren et al. 2006; Wood and Yezerinac 2006). Other studies underline that naturally divergent habitats also may cause differentiation in acoustic traits, as shown for the Little Greenbul (Slabberkoorn and Smith 2002). In the Green warbler ring species, a complex interplay of ecological differences, balancing between natural and sexual selection, has been invoked (Irwin 2000).

In this study, we recorded contact calls of the homogenously distributed Abyssinian white‐eye *Zosterops abyssinicus* across major parts of Kenya. This bird is a typical savannah species, common in large parts of East Africa at lower elevations, and generally found in large flocks (Zimmermann et al. 1996; BirdLife International, 2015). In contrast to this rather interconnected distribution setting, a large variety of closely related congenerics, i.e. *Z. poliogaster* and allies, occur restricted to mountain ranges (Mulwa et al. 2007). These mountain taxa have diverged into a variety of genetic lineages (Cox et al. 2014). Generally, the highland and lowland taxa of *Zosterops* do not co‐occur. However, at a few localities, both ecotypes can be found in sympatry (BirdLife International, 2015). In this study, we make use of this setup to study the effects of different distribution settings, geographic isolation and occurrence in sympatry with close relatives to understand the evolution of contact calls in *Z. abyssinicus*. We recorded and analyzed contact calls for populations sampled along a latitudinal gradient. Based on this study setup and 2795 contact calls recorded at 19 sites, we try to explore the following questions:

1. Are populations differentiated in contact call patterns despite the homogenous distribution of *Z. abyssinicus*?
2. Do latitude, longitude, or altitude have any impact on differentiation patterns of contact calls in *Z. abyssinicus*?
3. Does sympatry with congeners affect contact calls of *Z. abyssinicus*?

## Material and Methods

### Data collection

**Figure 1:**
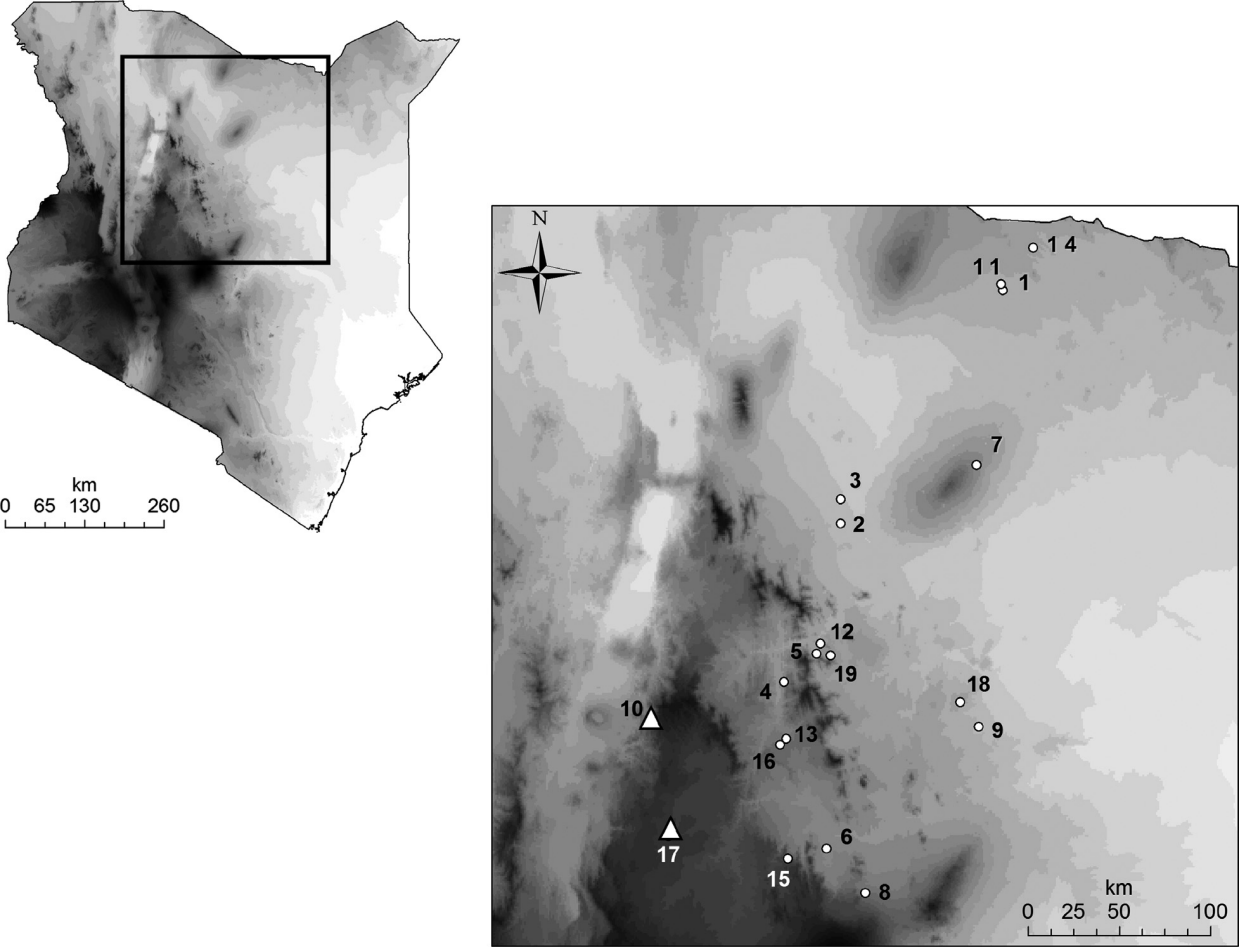
Map of Kenya showing the distribution of all localities, where contact calls of *Zosterops abyssinicus* were recorded. Circles represent populations without a congener, whereas triangles represent locations where *Z. abyssinicus* occurs in sympatry with a congeneric species. Numbers coincide with Table 1.

We recorded contact calls of the lowland bird species *Z. abyssinicus* at 19 sites (Fig. 1, Table 1). At two of these sites (Nairobi and the Thika coffee farm), *Z. abyssinicus* occurs sympatrically with taxa belonging to the highland species complex (i.e., the Montane white‐eye species *Z*. poliogaster). Contact calls were recorded during spring and summer 2013 and 2014 between 6:00 am and 6:00 pm for a period of 2 days per site using a Rhode NTG‐2 Dual Powered Directional Condenser Microphone. It was not possible to distinguish between male and female bird individuals. A digital Zoom‐H4 recorder was used to save the calls as stereo wav‐files. Contact calls of the birds were recorded with a distance of approximately five meters between the microphone and the target individual. *Zosterops abyssinicus* mostly emits contact calls in series and regular intervals; often several individuals call simultaneously. Birds are using such contact calls to persist as flocks when they are moving through the thicket (Kondo and Watanabe 2009); calls are further thought to have a function in mate recognition in the genus (Robertson 1996). As *Z. abyssinicus* occurs in flocks (sizes ranging from few individuals to some tens), our data set may contain some repeated recordings from same individuals. To minimize this, recordings were stopped after a maximum of 5 clear and loud calls, and the next recording was performed at another edge of the bird flock.

**Table 1:** Overview of all collected contact calls of *Zosterops abyssinicus*. Given is the name of geographic location, exact geographic coordinates (longitude and latitude), altitude, altitude classes, number of calls collected, cross‐taxon co‐occurrence situation, and the sampling date. The runnings number coincide with Figure 1

No	Location	Longitude	Latitude	Altitude	Altitude class	N	Co‐occurrence	Date
1	Chyulu Kibwezi	38°22′	3°27′	1110	Intermediate	166	Allopatric	02‐2013
2	Hunters Lodge	37°42′	2°12′	929	Intermediate	85	Allopatric	02‐2012
3	Machakos	37°42′	2°24′	942	Intermediate	301	Allopatric	02‐2012
4	Makuli Forest	37°14′	1°34′	1569	Highland	160	Allopatric	08‐2013
5	Masinga Dam	37°30′	1°48′	1663	Highland	36	Allopatric	08‐2014
6	Mt. Kasigau Rukanga	37°35′	0°52′	1014	Intermediate	34	Allopatric	08‐2014
7	Mtito Andei	38°09′	2°41′	758	Lowland	129	Allopatric	02‐2013
8	Mumoni Hills	37°54′	0°30′	617	Lowland	121	Allopatric	02‐2013
9	Mutito	38°10′	1°12′	699	Lowland	19	Allopatric	08‐2013
10	Nairobi	36°48′	1°16′	1674	Highland	41	Sympatric	02‐2013
11	Nzaui Rock	38°21′	3°30′	933	Intermediate	359	Allopatric	02‐2013
12	Oldonio Sabuk	37°32′	1°53′	1572	Highland	90	Allopatric	08‐2014
13	Sagana	37°15′	1°06′	1487	Highland	93	Allopatric	08‐2013
14	Taita Lowland Dembwa	38°37′	3°48′	604	Lowland	259	Allopatric	08‐2013
15	Taita Mwatate	37°16′	0°47′	1057	Intermediate	137	Allopatric	03‐2014
16	Thika Darcy	37°12′	1°03′	1447	Highland	249	Allopatric	08‐2014
17	Thika Coffee farm	36°57′	0°58′	1599	Highland	84	Sympatric	03‐2014
18	Wikililye	38°01′	1°24′	1088	Intermediate	353	Allopatric	08‐2013
19	Wothe	37°37′	1°47′	1134	Intermediate	79	Allopatric	08‐2014

Contact calls of high quality were further processed with the program PRAAT vers. 5.2.15 (Boersma 2002). Calls being affected by strong background noise or overlapping with other calls were excluded from further analyses. After deleting calls of bad quality, a total number of 2795 calls remained (with an average of 147 calls per site ± 107, ranging from 19 to 359 calls) (see Table 1).

For each call, we measured the following parameters: starting frequency (sometimes similar with lowest frequency), first peak (mostly similar with highest frequency), end frequency (mostly similar with lowest frequency), lowest and highest frequency, total duration of call (in seconds), and the range of frequencies (difference between the lowest and the highest frequency). A typical sonogram is displayed in Husemann et al. (2014b). Spectral analyses were performed blind to site. The spectrogram settings menu was used to adjust the range of frequencies (Hz) and the dynamic range (dB) depending background noise.

For comparative interspecific analyses, we included 1494 contact calls of populations of the Montane white‐eye complex (i.e., *Z. poliogaster*), with populations from nine Kenyan mountain sites taken from a previous study (Husemann et al. 2014b); data were collected and analyzed in the same ways as described above.

### Statistics

We compared contact calls among study sites using one‐way PERMANOVA (Anderson et al. 2001) based on the correlation matrix as implemented in Primer 7 (Primer‐E Ltd., Plymouth, UK). Study site and altitude (divided into lowland, intermediate altitude, and highlands) and the co‐occurrence with congeners (allopatric, sympatric) served as categorical variables. Euclidean distance‐based principle coordinates analysis (PCoA) was employed to differentiate between local populations. The two dominant PCoA axes explained 41 and 31% of variance, respectively. We used ordinary least squares regression to assess geographic trends in call structure. Additionally, spatial distance decay in call structure and call variability were assessed by Mantel correlation using Euclidean call and geographic distances in PAST 3.0 (Hammer et al. 2001; Hammer 2013); 5000 randomizations were performed to assess significance. Variability in contact calls among individuals was assessed by the CV (coefficient of variance) using 5000 bootstrap samples as implemented in PAST to estimate the respective confidence limits. To assess variability across sites, we calculated the coefficient according to Lloyd (CL) (Lloyd 1967), which reaches a value of unity in the case of a Poisson random distribution, indicating that variance and mean are independent.

We compared contact calls of sympatric and allopatric populations of *Z. abyssinicus* and *Z. poliogaster* using PCoA (Euclidean distances) based on *Z*‐transformed call parameters (*Z* = (*x* − *m*)/*s*,* x*: value, *m*: arithmetic mean of all values, *s*: respective standard deviation). Due to the high number of records from the allopatric populations of both species, which might bias the PCoA axes (>1200 in each species), we used 200 randomly chosen calls from each species for the analysis. This number is similar to the numbers of calls obtained from the sympatric populations of each species.

## Results

**Figure 2:**
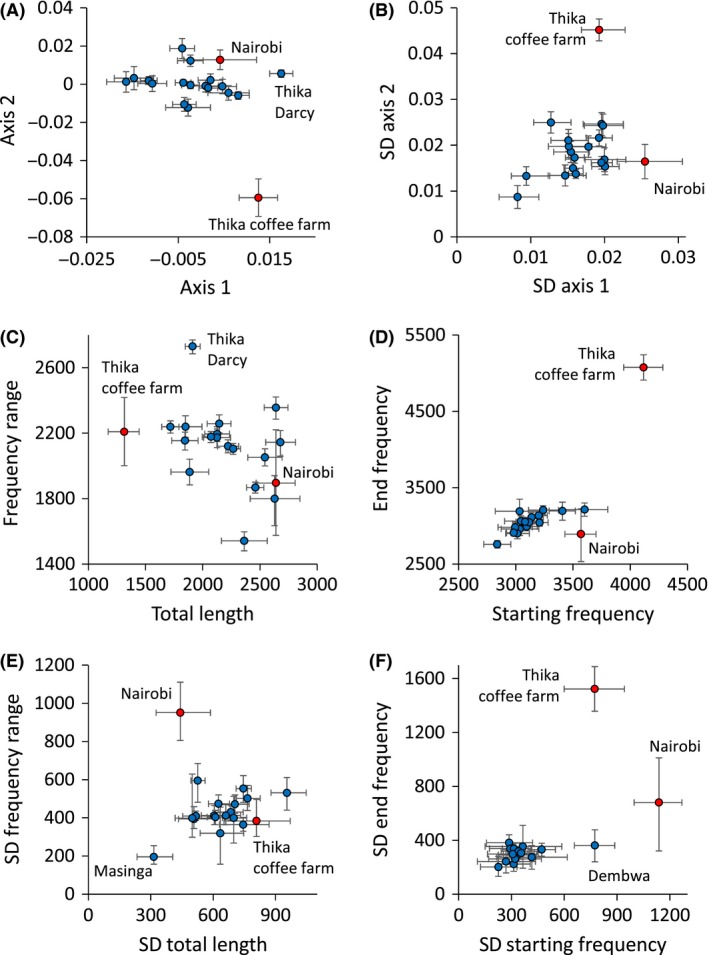
Averaged (±95% bootstrapped confidence limits) contact call characteristics of *Zosterops abyssinicus* along the first two principle coordinates axes (Euclidean distances) among the 19 study sites (A). Variability (standard deviation of the first two axes ± two bootstrapped 95% confidence limits) in contact calls (B). Blue: sites of allopatric co‐occurrences, with *Zosterops poliogaster*, red: sympatric sites. Total length – frequency range (C, E) and starting frequency – end frequency (D, F) plots (C, D: average values; E, F: respective standard deviations) separated the Thika coffee farm and less pronounced the Nairobi populations from all other populations. Blue: sites of allopatric co‐occurrences, with *P. poliogaster*, red: sympatric sites. Error bars denote 95% bootstrapped confidence limits.

We found high local variability of contact calls across all sites, in accordance with a Poisson random distribution. The Lloyd coefficients of all seven call parameters only marginally differed from unity (average 1.03 ± 0.01) demonstrating a large overlap in call patterns among allopatric sites (Fig. 2A). Nevertheless, despite of this high variability, PCoA (Fig. 2A) and PERMANOVA (Table 2) revealed subtle local dialects. Location explained 2.0% and co‐occurrence with the congeneric species 7% of the variation in contact call structure (Table 2).

**Table 2:** One‐way PERMANOVA detected subtle differences in *Zosterops abyssinicus* contact calls across the allopatric study sites, the altitudinal classes (lowland, intermediate, and highland), and the pattern of co‐occurrence with *Zosterops poliogaster* (allopatric, sympatric). *r* ^2^ refers to the variance explained by each factor

Factor	df	pseudo‐*F*	*P*	*r* ^2^
Site	16	48.3	<0.0001	0.02
Altitude	2	37.3	<0.0001	0.01
Co‐occurrence	1	198.5	<0.0001	0.07
*N* (allopatric)	2670			

**Figure 3:**
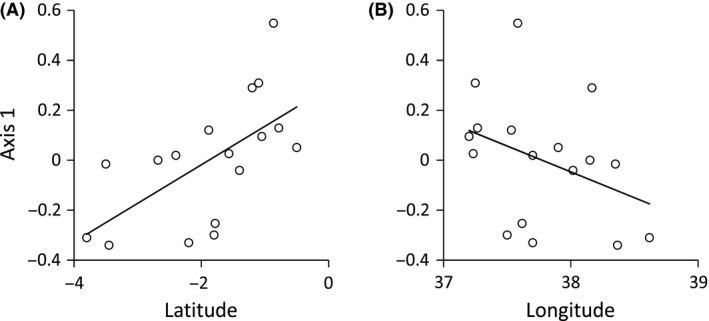
Latitudinal (A: linear OLS regression: *r* ^2^ = 0.38, *P*(*F*) = 0.01) and longitudinal (B: *r* ^2^ = 0.13, *P*(*F*) > 0.10) trends of contact call patterns of populations of *Zosterops abyssinicus* condensed to the first PCoA axis, exclusively based on populations without co‐occurrences with the highland taxa.

The subtle local differences in contact call structure represent a significant latitudinal (Fig. 3A) and a weak longitudinal (Fig. 3B) gradient. Consequently, Mantel tests based on averaged records per site identified a weak, but significant spatial distance decay in contact call structure (r = 0.03, *P* < 0.01). PERMANOVA revealed only a weak influence of altitude (Table 2).

**Figure 4:**
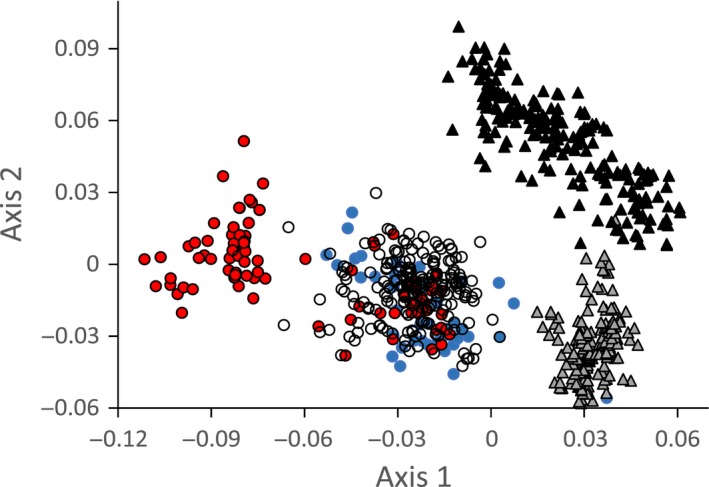
Principle coordinates analysis (Euclidean distances) separated the bird calls of *Zosterops abyssinicus* (circles) and *Zosterops poliogaster* (triangles). Within *Zosterops poliogaster* sympatric populations (gray triangles) differ from allopatric ones (black triangles). = Zosterops abyssinicus populations at allopatric sites (open circles) are homogeneous, while the sympatric population at Thika coffee farm (red circles) is divided into two parts. The sympatric Nairobi population of *Z. abyssinicus* clusters within the allopatric populations, but shows a higher variability (cf. Fig. 2).

To test for potential effects of sympatry with congeners on call characteristics we contrasted calls recorded in sympatric populations (i.e., Nairobi and Thika coffee farm) with those from allopatric populations (all other remaining 17 populations) and with similar data from the highland congeneric *Z. poliogaster* (Figs. 2, 4). The Thika coffee farm population clearly differed from populations at allopatric sites (Fig. 2A), while this was less obvious for the Nairobi population. The Thika coffee farm population differed particularly in total call length (Fig. 2C) and end frequency (Fig. 2D). However, both sympatric sites strongly differed in the variability of the calls (Fig. 2E and F). Contact calls at Thika coffee farm and in Nairobi were significantly more variable than at the allopatric locations (one‐way ANOVA: *F*_1,18_ = 6.8, *P* = 0.01). PCoA separated allopatric populations of both species (Fig. 4) and also distinguished the sympatric *Z. poliogaster* population from Nairobi. The sympatric Thika coffee farm population of *Z. abyssinicus* was subdivided: calls of one part of the population were similar to contact calls of the allopatric conspecific populations, whereas calls of the other part of the population clearly diverged from all other populations of both species (Fig. 4). In turn, the sympatric Nairobi population of *Z. abyssinicus* clustered within its conspecifics, yet had a significantly higher variance in call characteristics (Fig. 2).

## Discussion

### Call divergence despite a continuous distribution

Contact calls of the genus *Zosterops* are considered to be an important, species‐specific social signal facilitating flock structure maintenance and playing a role for mate recognition (Robertson 1996; Kondo and Watanabe 2009). Our data for *Z. abyssinicus* indicate weak, but detectable divergence in call characteristics despite the continuous distribution of the species. A prior study demonstrated a lack of genetic differentiation for the same species across a geographic range of more than 600 km (Habel et al. 2013). This suggests that local populations are interconnected and have high gene flow across East African savannahs. Thus, the question arises, which drivers may cause this call differentiation – despite the species′ panmictic occurrence?

Patterns and drivers of the evolution of call divergence have been analyzed for a variety of species, yet, most of them occurring in geographic separation (Kroodsma and Miller 1996). For example, the Montane white‐eye species complex (*Z. poliogaster* and relatives) occurs in geographically separated populations since a long time (cf. Cox et al. 2014), which has led to the accumulation of genetic (Habel et al. 2013, 2014; Husemann et al. 2015a), phenotypic (Borghesio and Ndanganga 1999; Habel et al. 2015) and bioacoustic differences (Habel et al. 2013; Husemann et al. 2014b). Such differentiation may occur in relatively small and isolated populations as a product of stochastic processes (e.g., Husemann et al. 2015a,b). However, our target study species has a continuous distribution across large stretches of relatively homogeneous habitat (i.e., savannah). Here, differences in local selective regimes due to diverging local environmental conditions and subsequent species′ adaptation may drive differentiation of call characteristics. This has already been suggested for a variety of other bird species, such as the Little greenbul *Andropadus virens* (Slabberkoorn and Smith 2002).

### Gradual call divergence despite high levels of gene flow

We observed a gradual divergence of call characteristics along latitudinal and longitudinal transects despite high levels of gene flow, which had been previously demonstrated for the species (Habel et al. 2013). This may be surprising considering the good flight abilities of the species and the lack of strong geographic barriers across the range, which should lead to a homogenization of the gene pool and prevent any differentiation (Zimmermann et al. 1996; Habel et al. 2013) (see above). Gradients in contact calls across homogeneous distributions were also found in the Australian silvereye *Zosterops lateralis* (Baker 2012) and in a variety of other homogeneously distributed species with acoustic communication (Ryan and Wilczynski 1991; Ryan et al. 1996; Irwin 2000; Nosil et al. 2005; Cole 2010). Such intraspecific acoustic gradients may result from gradually changing environmental conditions or simply from drift effects along the geographic range (cf. Cole 2010). As most of the *Z. abyssinicus* populations occur in large metapopulation networks, the first scenario – environmental gradients – might be the most plausible explanation, as drift should be counterbalanced by permanent exchange of individuals and gene flow and the existence in comparatively large local populations. Mate choice based on acoustic traits at a local level might finally support this pattern of bioacoustic gradients (see review by Gerhardt 2012). Alternatively, ecological differences may influence the balance between natural and sexual selection, which may lead to divergence, as suggested for the greenish warbler (Irwin 2000).

### Call divergence in sympatry

In a previous study, Husemann et al. (2014b) suggested that *Zosterops* may change call characteristics in response to the presence of a closely related, congeneric species. Here, we provide further support for acoustic character displacement in this genus, which may even have led to cascade reinforcement potentially explaining how reproductive isolation may have evolved in the group (Ortiz‐Barrientos et al. 2009). At the two sites where a congener co‐occurs with *Z. abyssinicus*, we detected a significantly higher variability in contact calls and a deviation of some call characteristics (Fig. 2). At least part of the sympatric population significantly deviated in call characteristics from allopatric populations (Fig. 2) – yet, the call traits in which the populations diverged differed depending on location. Intermediate call characteristics between *Z. abyssinicus* and *Z. poliogaster* calls found in the Nairobi population may be a product of occasional hybridization of both taxa or potential cross‐species learning. In contrast, call characteristics recorded at Thika strongly diverge from both, typical *Z. abyssinicus* and *Z. poliogaster* calls, and thus may be the result of character displacement, a mechanism maximizing the differences of signaling phenotypes to prevent hybridization in sympatry, when closely related species get into secondary contact (Coyne and Orr 1989, 1997; Kirkpatrick and Ravingne 2002; Bernasconi et al. 2004). This phenomenon has been demonstrated to be of importance in a variety of species including grasshoppers (Marshall and Cooley 2000; Tregenza et al. 2001), crickets (Panhuis et al. 2001), amphibians (Höbel and Gerhardt 2003; Lemmon 2009), and birds (Wallin 1986; Via 1999; Haavie et al. 2004; Kirschel et al. 2009). Hence, character displacement represents a common and important mechanism rapidly increasing differentiation in signaling traits between close relatives and may often ultimately facilitate speciation (Gavrilets 2000; Irwin 2000); similar mechanisms had been suggested to be important drivers in the radiation of white‐eyes in other regions or the world (Clegg et al. 2008; Moyle et al. 2009).

## Conclusions and critical data review

Our data indicate that even homogenously distributed taxa like *Z. abyssinicus* may diverge in acoustic traits over large areas. We suggest that slightly different environmental conditions may have led to the gradual differentiation in contact calls. Secondly, populations of the lowland *Z. abyssinicus* sympatric with highland congeners show strong call divergence – yet, traits shift in different directions, and most probably are driven by different factors. However, as each phenomenon is supported by only one single population, our interpretation of these data has to be treated with caution and will have to be confirmed in the future. Common garden experiments are needed to fully understand the importance of acoustic communication for species recognition in the genus and to understand the factors driving call divergence.

## Conflict of Interest

None declared.
